# Strategies used by gay male HIV serodiscordant couples to reduce the risk of HIV transmission from anal intercourse in three countries

**DOI:** 10.1002/jia2.25277

**Published:** 2019-04-15

**Authors:** Benjamin R Bavinton, Garrett P Prestage, Fengyi Jin, Nittaya Phanuphak, Beatriz Grinsztejn, Christopher K Fairley, David Baker, Jennifer Hoy, David J Templeton, Ban K Tee, Anthony Kelleher, Andrew E Grulich, the Opposites Attract Study Group

**Affiliations:** ^1^ The Kirby Institute UNSW Sydney Sydney Australia; ^2^ PREVENTION Thai Red Cross AIDS Research Centre Bangkok Thailand; ^3^ Evandro Chagas Institute of Clinical Research (IPEC) FIOCRUZ Rio de Janeiro Brazil; ^4^ Melbourne Sexual Health Centre Melbourne Australia; ^5^ Monash University Melbourne Australia; ^6^ East Sydney Doctors Sydney Australia; ^7^ The Alfred Hospital Melbourne Australia; ^8^ RPA Sexual Health Sydney Australia; ^9^ Centre Clinic Melbourne Australia

**Keywords:** serodiscordant couples, homosexual, gay men, men who have sex with men, HIV prevention, risk reduction strategies, sexual behaviour

## Abstract

**Introduction:**

There are few data about the range of strategies used to prevent sexual HIV transmission within gay male serodiscordant couples. We examined HIV prevention strategies used by such couples and compared differences between countries.

**Methods:**

*Opposites Attract* was a cohort study of male serodiscordant couples in Australia, Brazil and Thailand, from May 2014 (Australia) or May 2016 (Brazil/Thailand) to December 2016. At visits, HIV‐positive partners had viral load (VL) tested; HIV‐negative partners reported sexual behaviour and perceptions of their HIV‐positive partner's VL results. Within‐couple acts of condomless anal intercourse (CLAI) were categorized by strategy: condom‐protected, biomedically protected (undetectable VL and/or pre‐exposure prophylaxis [PrEP]), or not protected by either (HIV‐negative partners engaging in insertive CLAI, receptive CLAI with withdrawal, or receptive CLAI with ejaculation).

**Results:**

A total of 343 couples were included in this analysis (153 in Australia, 93 in Brazil and 97 in Thailand). Three‐quarters of HIV‐positive partners were consistently virally suppressed (<200 copies/mL) during follow‐up, and HIV‐negative partners had correct perceptions of their partner's VL result for 76.5% of tests. One‐third of HIV‐negative partners used daily PrEP during follow‐up. Over follow‐up, 73.8% of couples had CLAI. HIV‐negative partners reported 31,532 acts of anal intercourse with their HIV‐positive partner. Of these, 46.7% were protected by condoms, 48.6% by a biomedical strategy and 4.7% of acts were not protected by these strategies. Australian couples had fewer condom‐protected acts and a higher proportion of biomedically protected acts than Brazilian and Thai couples. Of the 1473 CLAI acts where the perceived VL was detectable/unknown and were not protected by PrEP (4.7% of all acts), two‐thirds (n = 983) were when the HIV‐negative partner was insertive (strategic positioning). Of the 490 acts when the HIV‐negative partner was receptive, 261 involved withdrawal and 280 involved ejaculation. Thus, <1% of acts were in the highest risk category of receptive CLAI with ejaculation.

**Conclusions:**

Couples used condoms, PrEP or perceived undetectable VL for prevention in the majority of anal intercourse acts. Only a very small proportion of events were not protected by these strategies. Variation between countries may reflect differences in access to HIV treatment, education, knowledge and attitudes.

## 1 Introduction

HIV‐negative partners in gay male serodiscordant couples are considered high risk for HIV infection 1, and it is often recommended that serodiscordant couples be a key focus of HIV prevention interventions 1, 2. Estimated proportions of new infections acquired from “steady” or “regular” relationships vary widely (e.g. 34% from “regular partners” in Australia; 32‐39% from “main partners” in Peru; 68% from “main partners” in the United States; and 74‐90% from “steady partners” in Amsterdam) 3, 4, 5, 6. In Australia, an incidence of 2.2 HIV infections per 100 person‐years among HIV‐negative men in serodiscordant regular partnerships has been described 7.

Serodiscordant couples use a range of strategies to prevent within‐couple HIV transmission. Condoms for anal intercourse have been promoted for several decades and are still, for many, the defining feature of “safe(r) sex.” Population‐level effectiveness estimates of consistent condom use range from 72 to >90%, depending on the methods of calculation used 8, 9, 10, 11. Many long‐term gay couples use condoms less often than for casual sex, as condomless anal intercourse (CLAI) is associated with greater relationship commitment, satisfaction, love, intimacy and trust 12, 13, 14, 15. Strategic positioning refers to the HIV‐negative partner exclusively taking the insertive position in CLAI 16, and has similar HIV risk compared with not having any CLAI among men having anal intercourse 17. Couples can use withdrawal before ejaculation to reduce risk, however, withdrawal has not been found to confer additional protection over CLAI with ejaculation 17. More recently, studies have demonstrated that pre‐exposure prophylaxis (PrEP) in HIV‐negative men is highly effective at reducing HIV risk provided adherence to the medication is high 18, 19, and has been recommended specifically for serodiscordant couples 20. Although uptake of PrEP among gay men is increasing rapidly in some countries including Australia 21, at a global scale it is still limited 22. Finally, “treatment as prevention,” or the reliance on undetectable VL to prevent transmission when having condomless sex, is highly effective for both heterosexual and gay male serodiscordant couples 23, 24, 25.

There has been little published research about the full *range* of HIV prevention strategies used by male serodiscordant couples. Studies typically focus on one or a small group of strategies 26, often include only a small number of couples 26, 27, or do not include both members of the couple 28. In a cohort study in Australia, Brazil and Thailand, we aimed to examine the strategies that gay male serodiscordant couples used to reduce within‐couple HIV transmission risk, and to compare differences between countries.

## 2 Methods

### 2.1 Procedures and participants

Detailed methods are published elsewhere 25, 29. Briefly, *Opposites Attract* was an observational prospective cohort study of gay male serodiscordant couples conducted through 13 clinics in Australia, and one clinic each in Rio de Janeiro, Brazil, and Bangkok, Thailand. Enrolment commenced on 7 May 2012 in Australia and 8 May 2014 in Brazil and Thailand, and continued to 31 March 2016. Follow‐up ceased for all couples on 31 December 2016. Individual couples were followed‐up from the time of their baseline visit until the end of the study or until they withdrew, became ineligible or were lost‐to‐follow‐up. At enrolment, written informed consent was obtained from all participants. Study information was provided to couples together or separately; consent procedures were conducted separately for the partners in each couple. The study was approved by human research ethics committees in each country 29.

The study recruited 358 male serodiscordant couples where: both partners were ≥18 years, couples reported at least monthly anal intercourse, and partners expected to be having sex with each other by the time of the HIV‐positive partner's next VL test. Couples were scheduled to attend at least two clinic visits per year. At each visit, HIV‐positive partners had a VL test, HIV‐negative partners had an HIV antibody test, and both partners completed an online computer‐assisted self‐interview (CASI) either at home (Australia) or in a private area of the clinic (Brazil and Thailand). Couples that enrolled but did not attend any follow‐up visits (n = 15) were excluded from this analysis.

### 2.2 Data collection and measures

Data were collected from clinics using standardized electronic case report forms on: antiretroviral therapy (ART) regimen and VL in the HIV‐positive partner, and HIV serology results in the HIV‐negative partner. Clinics reported the actual VL in RNA copies/mL if the result was “detectable,” and the test's lower limit of detection if “undetectable.” Viral suppression was defined as <200 copies/mL.

In the online CASIs, participants reported demographic information, sexual identity and length of time since ever first having sex together. HIV‐negative partners reported: sexual behaviour since last study visit both within the couple and with casual partners (including number of anal intercourse acts with various partner types, condom use, sexual positioning and the presence or absence of ejaculation during CLAI); their perceptions of their HIV‐positive partner's current and previous VL test result (“perceived VL”); and PrEP use. Their perception of the last VL test was used to define “perceived VL” for the period since the last questionnaire. “Daily PrEP” use was defined as using PrEP all or most days of the period since the last questionnaire. HIV‐negative partners reported the number range of acts for each category of anal intercourse (i.e. “none,” “one,” “two,” “three to five,” “six to 10,” “11 to 30,” “31 to 50” and “over 50”). The mid‐point of the range was taken as the number of acts in that category in the previous period, except for “over 50” which was taken as 51 acts. Acts were classified according to HIV prevention strategy: condom‐protected acts; and biomedically protected CLAI, which included perceived undetectable VL (UVL) and daily PrEP use. The remaining acts were not protected by condoms, perceived UVL or daily PrEP. These CLAI acts were further classified into those where the HIV‐negative partner was insertive (strategic positioning), where the HIV‐negative partner was receptive but the HIV‐positive partner withdrew before ejaculation, and where the HIV‐negative partner was receptive and ejaculation occurred inside the rectum.

### 2.3 Statistical analysis

Data were analysed in Stata 14.1 (Stata Corporation, Texas, USA). Significant differences between countries were determined by univariable chi‐square tests or analysis of variance tests. Univariable logistic regression was used to determine significant differences between countries in perceptions of VL test results and the proportions of anal intercourse acts for various categories.

## 3 Results

Couple characteristics have been previously reported 25. Briefly, mean age at enrolment was 36 years (±10 years; Table 1). Australian participants were older and more likely to be white/Caucasian than Brazilian and Thai participants (*p* < 0.001). About half of participants were university educated. The majority self‐identified as “gay.” Nearly half (42.3%) of the couples first had sex with each other within the twelve months prior to enrolment; Australian couples had been having sex with each other longer than Brazilian and Thai couples (*p* = 0.003).

**Table 1:** Characteristics of HIV‐negative partners, HIV‐positive partners and couples: Total sample and by country

	Total (n = 343)	Australia (n = 153)	Brazil (n = 93)	Thailand (n = 97)	*p*‐value^a^
Age at baseline – mean (standard deviation)					
HIV‐negative partner	35.8 (10.1)	39.8 (10.1)	34.7 (10.0)	30.8 (7.4)	<0.001
HIV‐positive partner	35.9 (10.4)	40.6 (10.4)	35.4 (9.2)	29.0 (7.0)	<0.001
Ethnicity – n (%)					
HIV‐negative partner					
White/Caucasian	170 (49.6)	134 (87.6)	35 (37.6)	1 (1.0)	<0.001
Asian	111 (32.4)	13 (8.5)	2 (2.2)	96 (99.0)	
Black	15 (4.4)	0 (0.0)	15 (16.3)	0 (0.0)	
Indigenous	3 (0.9)	1 (0.7)	2 (2.2)	0 (0.0)	
Other and/or mixed	44 (12.8)	5 (3.3)	39 (41.9)	0 (0.0)	
HIV‐positive partner					
White/Caucasian	167 (48.7)	123 (80.4)	43 (46.2)	1 (1.0)	<0.001
Asian	116 (33.8)	18 (11.8)	2 (2.2)	96 (99.0)	
Black	16 (4.7)	1 (0.7)	15 (16.1)	0 (0.0)	
Indigenous	2 (0.6)	2 (1.3)	0 (0.0)	0 (0.0)	
Other and/or mixed	42 (12.2)	9 (5.9)	33 (35.5)	0 (0.0)	
University education – n (%)					
HIV‐negative partner	188 (54.8)	82 (53.6)	42 (45.2)	64 (66.0)	<0.001
HIV‐positive partner	168 (49.0)	70 (45.8)	38 (40.9)	60 (61.9)	<0.001
Gay sexual identity – n (%)					
HIV‐negative partner	315 (91.8)	149 (97.4)	83 (89.3)	83 (85.6)	<0.001
HIV‐positive partner	321 (93.6)	151 (98.7)	81 (87.1)	89 (91.8)	0.006
Length of time since first sex together at baseline – n (%)^b^					
Less than 12 months	145 (42.3)	50 (32.7)	50 (53.8)	45 (46.4)	0.003
One to five years	118 (34.4)	54 (35.3)	29 (31.2)	35 (36.1)	
More than five years	80 (23.3)	49 (32.0)	14 (15.1)	17 (17.5)	
Antiretroviral treatment (ART) over follow‐up – n (%)^c^					
Never took ART	6 (1.8)	1 (0.7)	2 (2.2)	3 (3.1)	<0.001
Started ART during follow‐up	85 (24.8)	22 (14.4)	18 (19.4)	45 (46.4)	
Always on ART	252 (73.5)	130 (85.0)	73 (78.5)	49 (50.5)	
Viral load consistently <200 copies/mL over follow‐up – n (%)^c^	258 (75.2)	130 (85.0)	75 (80.7)	53 (54.6)	<0.001
Any use of daily PrEP over follow‐up – n (%)^b^	114 (33.2)	41 (26.8)	37 (39.8)	36 (37.1)	0.070
Any condomless anal intercourse (CLAI) within couples over follow‐up – n (%)^b^	253 (73.8)	136 (88.9)	64 (68.9)	53 (54.6)	<0.001

ART, antiretroviral therapy; CLAI, condomless anal intercourse.

^a^Statistical differences between countries were determined with chi‐square tests for categorical variables and analysis of variance for continuous variables; ^b^as reported by the HIV‐negative partner; ^c^as reported by the HIV‐positive partner.

Median per couple follow‐up was 1.7 years (interquartile range (IQR)=0.9‐2.2; Australia = 2.0 years (1.1‐3.2), Brazil = 1.5 years (1.0‐2.1), Thailand = 0.9 years (0.7‐1.8)), and there was a total of 588.4 couple‐years under observation. One‐third of the HIV‐negative partners took daily PrEP at some point during follow‐up. Of the 114 HIV‐negative partners who took daily PrEP during follow‐up, 38 (33.3%) took it for the entirety of follow‐up; 46 (40.4%) began PrEP during follow‐up and then stayed on it consistently; 9 (7.9%) began PrEP but subsequently stopped; 7 (6.1%) started it, had a break of at least one period between visits, then started it again; 6 (5.3%) were always on PrEP but had mixed patterns of adherence; and the remaining 8 (7.0%) reported varying usage patterns. Across follow‐up, there was no association between PrEP use and HIV‐positive partner's VL among the HIV‐negative partners who reported any PrEP use (*p* = 0.256). Nearly three‐quarters of HIV‐positive partners took ART throughout, while one‐quarter started ART during the study, and 1.8% did not take ART at any time. Similarly, three‐quarters had consistent virological suppression with VL of <200 copies/mL, 19.5% had VL that started ≥200 but then became consistently <200 copies/mL thereafter, 3.2% had VL variably under and over 200 copies/mL, and 2.0% had VL consistently ≥200 copies/mL. Thai participants were less likely to have been on ART and have consistent VL <200 copies/mL for the entirety of follow‐up (*p* < 0.001). All HIV‐negative partners reported within‐couple anal intercourse during follow‐up, and three‐quarters (73.8%) of HIV‐negative partners reported some CLAI with their study partner. More Australian couples reported within‐couple CLAI (88.9%) than those in Brazil (68.8%) and Thailand (54.6%; *p* < 0.001).

All available VL test results were compared to the HIV‐negative partner's perception of his HIV‐positive partner's VL. Most (76.5%) perceptions were “correct” when compared to VL as measured by pathology testing (Table 2): 71.2% perceived the result to be undetectable when it was <200 copies/mL, while 5.3% perceived the result to be detectable when it was >200 copies/mL. Of the 419 “not correct” perceptions, over two‐thirds (n = 287) were because the HIV‐negative partner believed the VL to be detectable when it was actually <200 copies/mL. Overall, 6.6% of perceptions were when HIV‐negative partners reported they did not know the result, and only 0.8% of perceptions (15 test results) were when the HIV‐negative partner believed the VL to be undetectable when it was actually >200 copies/mL. A higher proportion of HIV‐negative partners from Australia (90.4%) and Brazil (83.0%) had “correct” perceptions compared to those in Thailand (25.7%; *p* < 0.001).

**Table 2:** “Correct” and “not correct” HIV‐negative partner perceptions of HIV‐positive partner's viral load tests conducted at each visit

	Total^a^ (n = 1780)	Australia (n = 1017)	Brazil (n = 429)	Thailand (n = 334)
Perceived undetectable viral load, actually <200 copies (“correct”)	1267 (71.2)	895 (88.0)	335 (78.1)^b^	37 (11.1)^b^
Perceived detectable viral load, actually ≥200 copies (“correct”)	94 (5.3)	24 (2.4)	21 (4.9)^c^	49 (14.7)^b^
Did not know viral load (“not correct”)	117 (6.6)	38 (3.7)	34 (7.9)^b^	45 (13.5)^b^
Perceived detectable viral load, actually <200 copies (“not correct”)	287 (16.1)	50 (4.9)	34 (7.9)^c^	203 (60.8)^b^
Perceived undetectable viral load, actually ≥200 copies (“not correct”)	15 (0.8)	10 (1.0)	5 (1.2)	0 (0.0)

^a^Data on perceived viral load were missing for 56 visits (missed visit, n = 30; attended visit but did not complete questionnaire, n = 26); ^b^denotes significant differences between countries at the *p* < 0.001 level using univariable logistic regression, with Australia as the referent category; ^c^denotes significant differences between countries at the *p* < 0.05 level using univariable logistic regression, with Australia as the referent category.

**Figure 1:**
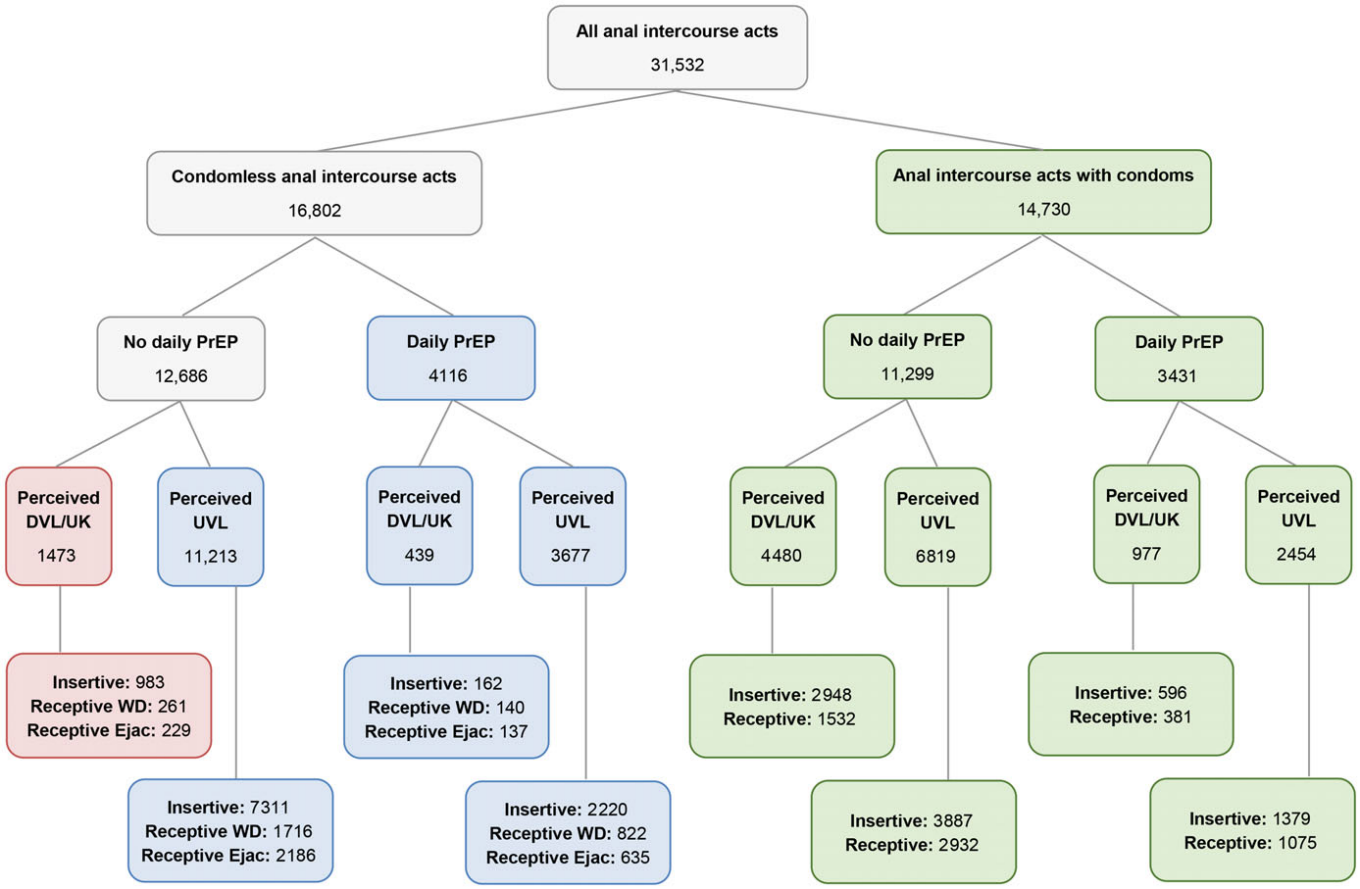
Mutually exclusive classification of 31,532 acts of anal intercourse within couples as reported by HIV‐negative partners, by HIV prevention strategy, during follow‐up in the Opposites Attract study. AI, anal intercourse; CLAI, condomless anal intercourse; Daily PrEP, PrEP use most or all days of the previous period; DVL/UK, detectable or unknown viral load; Ejac, ejaculation; PrEP, pre‐exposure prophylaxis; UVL, undetectable viral load; WD, withdrawal.

Over follow‐up, HIV‐negative partners reported a total of 31,532 acts of anal intercourse with their HIV‐positive study partner (Figure 1): 16,799 (53.2%) in Australia, 10,240 (32.5%) in Brazil and 4499 (14.3%) in Thailand. In total, 19,486 (61.8%) of anal intercourse acts were when the HIV‐negative partner was in the insertive position (61.0% in Australia, 58.5% in Brazil and 72.5% in Thailand). Just under half (46.7%) of the anal intercourse acts were protected by condoms, 48.6% by a biomedical strategy (perceived UVL and/or daily PrEP) and 4.7% of acts were not protected by either of these (Table 3). Higher proportions of acts were protected by condoms in Brazil and Thailand than in Australia (*p* < 0.001). Almost two‐thirds (63.0%) of the acts with condoms were also when the HIV‐positive partner was perceived to have UVL; this proportion was substantially lower in Thailand (7.1%) compared to > 80% in Australia and Brazil (*p* < 0.001). In Australia, 68.9% of acts were biomedically protected, while only 34.7% and 4.7% were biomedically protected in Brazil and Thailand respectively (*p* < 0.001). Within the biomedically protected acts, most acts were protected by perceived UVL only (73.1%) or perceived UVL plus PrEP (24.0%). Fewer acts in Brazil and Thailand were protected by perceived UVL than in Australia (*p* < 0.001). Within the acts protected by condoms and/or biomedical strategies, couples used condoms, PrEP or UVL only for 16,132 acts (53.7%) and some combination of these in 13,927 acts (46.3%). The proportion using a combination was 35.2% in Australia, 71.6% in Brazil and 27.4% in Thailand (*p* < 0.001). The proportion of acts not protected by either condoms or a biomedical strategy was higher in Thailand (10.4%, *p* < 0.001). Two‐thirds of these “not protected” acts were when the HIV‐negative partner was insertive, while receptive acts with ejaculation and withdrawal before ejaculation were evenly distributed (17.7% and 15.5% respectively). HIV‐negative partners in Thailand had a higher proportion of insertive “not protected” acts (91.5%) compared to Australia (52.9%) and Brazil (65.4%; *p* < 0.001).

**Table 3:** Acts of anal intercourse within couples as reported by HIV‐negative partners by HIV prevention strategy, total and by country

	Total (n = 31,532)	Australia (n = 16,799)	Brazil (n = 10,240)	Thailand (n = 4499)
Condom‐protected AI – n (%)	**14,730 (46.7)**	**4408 (26.2)**	**6506 (63.5)** a	**3817 (84.8)** a
Condoms only	4480 (30.4)	725 (16.4)	1028 (15.8)	2728 (71.5)^a^
Condoms and daily PrEP	977 (6.6)	5 (0.1)	155 (2.4)^a^	817 (21.4)^a^
Condoms and perceived UVL	6819 (46.3)	3355 (76.1)	3234 (49.7)^a^	230 (6.0)^a^
Condoms, perceived UVL and daily PrEP	2454 (16.7)	323 (7.3)	2089 (32.1)^a^	42 (1.1)^a^
Biomedically protected CLAI – n (%)	**15,329 (48.6)**	**11,571 (68.9)**	**3549 (34.7)** a	**213 (4.7)** a
Daily PrEP only	439 (2.9)	49 (0.4)	251 (7.1)^a^	140 (65.7)^a^
Perceived UVL only	11,213 (73.1)	9582 (82.8)	1575 (44.4)^a^	57 (26.8)^a^
Perceived UVL and daily PrEP	3677 (24.0)	1940 (16.8)	1723 (48.5)^a^	16 (7.5)^a^
Neither condom‐ or biomedically protected AI – n (%)	**1473 (4.7)**	**820 (4.9)**	**185 (1.8)** a	**469 (10.4)** a
Insertive (strategic positioning)	983 (66.7)	434 (52.9)	121 (65.4)^b^	429 (91.5)^a^
Receptive withdrawal	261 (17.7)	167 (20.4)	63 (34.1)^a^	31 (6.6)^a^
Receptive ejaculation	229 (15.5)	219 (26.7)	1 (0.5)^a^	9 (1.9)^a^

Percentages in bold use the column total as the denominator; percentages in normal type use the number of acts in each of the three main categories as the denominator (i.e. “condom‐protected AI,” “biomedically protected CLAI” and “neither condom‐ or biomedically protected AI”). AI, anal intercourse; CLAI, condomless anal intercourse; PrEP, pre‐exposure prophylaxis; UVL, undetectable viral load.

^a^Denotes significant differences between countries at the *p* < 0.001 level using univariable logistic regression, with Australia as the referent category; ^b^denotes significant differences between countries at the *p* < 0.01 level using univariable logistic regression, with Australia as the referent category.

There were 490 acts (1.6% of all anal intercourse acts) of receptive CLAI when the HIV‐positive partner was perceived to have detectable or unknown VL and when the HIV‐negative partner was not taking daily PrEP: 261 of these (53.3%; or 0.8% of all anal intercourse acts) involved withdrawal before ejaculation and 229 (46.7%; or 0.7% of all anal intercourse acts) involved ejaculation. Although these 490 acts occurred when the HIV‐positive partner was *perceived* to have detectable or unknown VL, pathology testing at the previous visit showed that 21 acts (4.2%; or 0.07% of all anal intercourse acts) were when the HIV‐positive partner actually had VL ≥200 copies/mL (18 with withdrawal, and 3 with ejaculation). All these acts occurred when the actual VL was greater than 1000 copies/mL.

## 4 Discussion

Men in these serodiscordant couples used a variety of strategies to reduce the risk of HIV transmission while having anal intercourse in their relationships. One‐quarter of the couples practiced consistent condom use and just under half of the reported acts of anal intercourse were condom‐protected. This study was conducted during a time of momentous change in HIV prevention internationally. High‐profile research evidence regarding TasP and PrEP was released throughout the course of the study, and consequently, individual and community notions of what constituted “safe(r) sex” among gay men were dynamic. Thus, CLAI was common, with three‐quarters of couples engaging in at least some CLAI during follow‐up, higher than estimates reported in previous studies of male serodiscordant couples 7, 26, 27, 30, 31. Nearly half of anal intercourse acts were perceived to be protected by a biomedical strategy. Within the acts protected by condoms and/or biomedical prevention, couples used either condoms, PrEP or UVL for over half of acts, while they used a combination of these for 46% of acts. There were several important differences between the three countries. Australian couples reported lower condom use and greater reliance on biomedical strategies, mainly perceived UVL. Couples in both Brazil and Thailand relied more heavily on condoms, and very few acts of anal intercourse among Thai couples were perceived to be protected by biomedical strategies. While less than five percent of anal intercourse acts were not protected by condoms or a biomedical strategy in Australia and Brazil, ten percent were in this category in Thailand, primarily due to the lower level of HIV treatment as well as “correct” knowledge of partners’ VL in Thailand.

Despite the perception of male serodiscordant couples as very high risk 1, these data suggest that for the most part, HIV transmission risk in these couples was low. Indeed, as previously reported, there were no phylogenetically linked within‐couple transmissions in this cohort 25. Less than 5% of all anal sex acts were neither protected by condoms nor by a biomedical strategy. Although it must be acknowledged that condoms, PrEP and TasP all rely on adherence and correct use, we found high levels of adherence to both PrEP and ART in those taking them 25. Within the acts not protected by condoms or biomedical prevention, two‐thirds had reduced risk due to the HIV‐negative partner being insertive (strategic positioning). Of the receptive acts, less than one percent involved ejaculation inside the rectum, and of this already small number of acts, almost all were when the HIV‐positive partner was highly likely to be virally suppressed, and thus actually not high risk. However, while they were few, all of the receptive “not protected” CLAI acts (with and without ejaculation) occurred when actual VLs were high.

Several factors may account for some differences between countries. First, among individual gay men in serodiscordant relationships, there may be differences in understanding and awareness of VL results, even though routine VL monitoring has been standard in each country for many years. At a population level, there may be lower HIV research literacy among men in Brazil and Thailand as compared to Australia, and related to this, there are likely to be differences in the ways that individuals incorporate new research findings about VL and prevention into their sexual lives. Although all HIV‐positive study participants had their VL tested at baseline, it should be noted that Thai guidelines state that VL testing should commence at least six months after ART initiation 32, which may partially account for the lower levels of knowledge among HIV‐negative partners of their HIV‐positive partner's VL test results. If couples decide to rely on TasP within relationships, they should be encouraged to discuss VL results openly with each other to ensure that decisions are being made with the best possible information 33. This may particularly be the case in Thailand, where higher proportions of HIV‐negative partners did not know their partner's VL test results or had “not correct” perceptions. However, a challenge for the Thai setting is that VL monitoring is recommended only annually 32. Furthermore, reliance on TasP will be limited by serostatus awareness and disclosure of serostatus within couples, both of which appear low in Brazil 34, 35. Second, there appears to be more limited dialogue in Brazil and Thailand about TasP among clinicians and health care workers, despite the three countries’ ART guidelines moving to treatment for all HIV‐positive patients 32, 36, 37. Additionally, there may be differences in the level of support from clinicians for UVL as a prevention strategy and consequently, varying degrees to which research findings are shared openly with patients. Third, there is likely to be a greater level of community awareness of VL generally in the Australian gay community, and more specifically of TasP, where there has been relatively widespread community dialogue about these topics for several years, initially spurred by the publication of the so‐called “Swiss Statement” in 2008 38. More recently, starting in 2012, Australia has seen several large‐scale community education campaigns targeting HIV‐positive gay men to consider early treatment, and targeting all gay men with explicit messages about the reduced risk of transmission when VL is undetectable (for example, see www.endinghiv.org). To our knowledge, these kinds of campaigns and gay community dialogue have not yet occurred in Brazil or Thailand. Although all study participants were kept updated throughout follow‐up on the latest TasP science, this information was not further reinforced by community dialogue outside of the study in Brazil and Thailand.

These data indicate that future studies exploring transmission risk in serodiscordant couples will need to account for the increasing range, and overlapping nature, of HIV prevention strategies. It may no longer be possible to separate and analyse the effects of a single strategy, such as TasP or PrEP, within this population. Future studies should ensure that detailed data are collected about all possible risk reduction methods. If data are simply not collected on certain strategies, the effects of the strategies that are being measured may be overestimated or confounded. For example, if a study focusing on undetectable VL did not also assess PrEP use, it may be concluded that TasP was solely responsible for HIV risk reductions when in fact it may have been due to PrEP or the combination of both. Future studies could also collect data on whether risk reduction methods were being used intentionally to prevent transmission or for other reasons (such as simply preferring one or other sexual position). Furthermore, studies of transmission risk in male serodiscordant couples will likely need very large sample sizes to be powered appropriately, or may need to target and recruit only genuinely high‐risk serodiscordant couples. This is likely to be challenging. With increases in TasP and PrEP in gay and bisexual populations, such “highest‐risk” couples (at least those linked to care within clinics) may become rare, especially as countries prioritize achieving the UNAIDS “90‐90‐90” targets 39. Indeed, in New South Wales, the Australian state where nearly twenty percent of *Opposites Attract* couples were enrolled, the 90‐90‐90 targets were achieved by end 2016 40, and 94% of all people diagnosed across the state in the first half of 2017 had initiated ART within three months of diagnosis 41. Time to ART initiation has also decreased markedly in Melbourne, Victoria, where 23.0% of couples were enrolled 42. The eligibility requirement that couples have at least some CLAI with each other has been used in other studies of serodiscordant couples 43, but this measure may no longer be sufficient to identify “high risk” 31, 44. Finally, previous analysis determined that risk is highest in the first year of a gay male serodiscordant relationship 7. This study has demonstrated that recruitment of couples who have been having sex for shorter durations is possible; future studies of serodiscordant couples should ensure that newer couples are targeted for recruitment, ideally within weeks of partnership formation.

These data raise important implications for HIV prevention interventions with serodiscordant couples. Clearly, many couples use multiple strategies to reduce risk. Modelling has suggested that over a 10‐year time‐scale, ART and condoms must be used together (or in combination with other strategies) to keep risk below 10% 45. However, over half of anal intercourse acts in our data were protected by one strategy only (e.g. 30% of the acts with condoms were protected only by condoms, and 76% of the biomedically protected CLAI acts were protected only by perceived undetectable VL *or* PrEP). Should serodiscordant couples be encouraged to use multiple prevention methods, or is it sufficient to encourage just one? What might be acceptable to couples? In any case, attention should be paid to those couples currently not employing any strategy to reduce transmission risk, especially those having CLAI when the HIV‐positive partner has high VL. Better education about TasP (particularly in Brazil and Thailand), greater access to PrEP, and education about the recommendation to use either condoms or PrEP during the first six months of treatment initiation 46 are all required. Another key issue for HIV education is that for many HIV‐negative men in serodiscordant couples, HIV risk from casual partners is likely to be higher than that from within the relationship 1. Couples’ interventions, by their nature, tend to focus on risk within relationships 2, 47. However, studies have shown high proportions of non‐monogamy and CLAI with casual partners among HIV‐negative partners in male serodiscordant couples 25, 48. Additionally, the *Opposites Attract* and *PARTNER* studies found no within‐couple HIV transmissions and showed that all incident infections came from casual partners 24, 25. Educational materials targeting men who have casual sex may not be perceived by men in couples as being relevant to them, even when one or both members of the couple have casual sex. This may especially be the case for so‐called “monogamish” couples 49 who only have sex with casual partners together. Therefore, couples’ interventions, at least those focused on gay men, should incorporate discussion of risk from casual partners.

Our analysis had some limitations. HIV‐negative partners’ estimates of the number of anal intercourse acts may not be correct due to the recall issues inherent in self‐reported data. Furthermore, the exact number of acts was estimated as the mid‐point of a range, whereas for some participants having over 50 acts, the number might be underestimated. While there may be some inaccuracies by taking this approach, it must be noted that asking participants to report exact numbers of anal sex acts is also challenging and likely to result in some inaccuracy. Accuracy may increase with the use of shorter recall periods (for example, weekly sexual diaries entered on mobile devices), but this must be balanced against the burden placed on participants, especially in studies running over several years such as this one. The couples may not be truly representative of all male serodiscordant couples in the three participating countries: couples were drawn from urban locations; they were predominantly recruited through clinics and thus were likely to be connected to care; participants were provided with education on HIV transmissions risk upon enrolment; and couples not having anal sex with each other at least once per month were excluded. On‐demand PrEP 50, post‐exposure prophylaxis (PEP) and condom breakage were not incorporated into the anal sex acts analysis because it was not possible to determine which acts occurred precisely when these were present. VL results from pathology at the previous visit were assumed to cover the entire subsequent period. While it is possible the VL could have changed throughout the period, we found such cases to be rare. Knowledge of VL results may have been impacted by the fact that participants in Australia completed their surveys at home, whereas those in Brazil and Thailand completed them at the clinic. However, participants were instructed to complete the questionnaires privately. Finally, the surveys did not collect data about whether or not the behaviours reported were intentionally being used to prevent HIV transmission.

## 5 Conclusions

Studies have now confirmed that transmission risk is low when HIV‐positive partners have undetectable VL 23, 24, 25. Many men in the couples enrolled in our study relied on undetectable VL to reduce transmission, along with other strategies such as condom use, PrEP, strategic positioning and withdrawal. Only a very small proportion of anal intercourse acts within the serodiscordant couples in *Opposites Attract* were not protected by any strategy. Our data showed large differences in couples from Australia, Brazil and Thailand, reflecting potential differences in HIV treatment, culture, education, knowledge and attitudes, and impacting upon the implications for HIV prevention in each setting.

## Competing interest

None declared.

## Authors’ contributions

AEG, GPP and FJ conceived of the study with input from CKF and AK. BRB, AEG, GPP and FJ designed the study and wrote the protocol with input from all authors. BRB, GPP, AEG and FJ designed the surveys and data collection tools. NP, BG, CKF, DJT, JH and BKT oversaw recruitment of couples at clinical sites. BRB analysed the data and drafted the manuscript. All authors contributed to the interpretation of results and approved the manuscript.
